# Cinderella: Attacking the Servant Problem

**Published:** 1919-02-08

**Authors:** 


### CINDERELLA.

### Attacking the Servant Problem.

By A SOCIAL WORKER.

THE idea of a College of Nursing would greatly have surprised Mrs. Gamp. The notion that the fool of the family might best be left in charge of domestic affairs when the promising daughters have gone to make their way in the world still lingers, though, as preventive medicine has assumed importance, the idea that management and maintenance are better than disaster and renewal has entered—or shall we say re-entered?—the [illegible]ish home.

### THE EVOLUTION OF THE HANDMAID.

There was a time when silver saucepans were used, for ladies cooked in them, and when priceless porcelain appeared on the tea-table instead of on the mantelpiece or over the door. There was a time—further back, certainly—when the family raiment had to go through every process from sheep-shearing to the last button in the home, and the family food had to originate in the clearing of forest to sow grain. The farmer and the miller and the tailor and other specialists lessened the industries of home till they became reduced to cleansing and cooking, and a vast accretion of bought ornaments and furniture and the fashion of much flower decoration made an immense amount of unneeded work. The house-ladies were not—perhaps could not be—much interested in such duties, and relegated them more and more to paid handmaidens, whom it was no one's care or business to instruct or even to supervise. "The house kept itself, and we kept a page," describes many a ménage as well as that of David Copperfield and his Dora. Then came improved education and widened choice of work and a growing distaste for "domestic service," and then the war with consequences in this direction which are familiar to us all.

We have lately seen an exaltation of Mothercraft; its praise is in the newspapers. We have seen doctor and surgeon respected not beyond their due, but in a manner that makes an amazing contrast with, for instance, the squire's condescension to the "apothecary fellow" in Mr. Lynn Linton's novel of not more than fifty years ago. And we have seen the profession of nursing take its present place, with the result that, as must always be the case, demands ever more urgent are made for the adequate training of aspirants to membership.

### THE AIM OF THE RESPECTABLE MOTHER.

But home is the point of leverage for the working life and the first school for the child, the place where the nerve-centres receive, for good or ill, their first training. However much its crafts may be delegated to specialists—and we begin to see how their banishment tends to loss of vital interest—the place itself remains and upon its atmosphere depends who shall say how much of the mental and physical health, happiness, and character of those who live in it? In "working-class" homes the mother is truly a "pivotal" person. But if it is good that there should be women possessed of leisure to follow professions or cultivate art or science or the service of the community the daily labour of their larger homes must be done by others, and domestic service is not merely an occupation artificially created for the benefit of the idle rich. Through many generations it was regarded by mothers of the poorer classes as the best of openings for their girls. During the most important years of youth they were well housed and abundantly fed, and taught domestic science under conditions impossible in their own homes, with great gain to their health of body. Indeed, it was often painful to see the falling-off in looks of girls after marriage or if circumstances had compelled them to go home. "Good" service was the aim of the respectable mother, work in the homes of gentlepeople under well-trained and well-principled upper servants, and a mistress careful of the ways of her own family and exacting as to the conduct of her maids and men.

### THE DRAWBACKS OF DOMESTIC SERVICE.

Such places are unfortunately few now compared to the mass of middle-class houses keeping one maid or two, and of hotels, clubs, boarding-houses, etc., where the personal character of employees does not receive the same anxious inquiry. Working-class mothers are sometimes reproached with unwillingness to send their young daughters into service where they must "live in," but a good mother often fears that the child may pass her days and nights in the constant companionship of a drunken, immoral, or dishonest fellow-servant older than herself. Consequently she goes to some other occupation and probably does not leave this again, unless, like dressmaking, it paves the way to a good position in service. Two other drawbacks now strongly felt are, of course, the want of outdoor air and the long hours required in some houses. To be on duty from 6 A.M. till 11 or 12 P.M. may be exceptional, but it does occur and cannot be wholesome from any point of view.

### AN ASSOCIATION OF "HOUSEKEEPERS."

The law of demand and supply, meantime, is working in the interests of the employees, and raising rates of pay and increasing hours for outing and holidays. But this does not tend to raise the status of a profession or increase the skill of the workers. The war has done more in this direction by the mere fact that it has shown the wastefulness of ignorant misuse of material and property. It is truly refreshing, therefore, to find some signs that the "servant" problem will be attacked from a new angle in the new world and the old. In Calgary, Canada, an interesting example has been given by the local "housekeepers," to use their self-chosen title, who have formed themselves into an association demanding certain rights and offering qualified service in return. The conditions comprised: A ten-hours' week-day and six hours' Sunday work-time, overtime to be paid at 15 cents an hour, the employee to be referred to as a "housekeeper," and addressed as Miss——, to use the front door if desired, and have the use of a room once in the week in which to receive friends, with due consideration as to quiet in the house, disputes to be referred to an arbitration committee which should have discretionary powers to suspend the member or black-list the employer. Some alarm was naturally felt by the housewives of Calgary. But silence fell, and after some time inquiry revealed a strange fact. When it came to painting the other side of the shield, where was the skill that was to qualify the "certified housekeeper?" The serving maidens of Calgary, when last heard of, were still being addressed by their Christian names and using the back door, but a regular system of lectures in cooking, canning, sanitation, laundry, and all the rest of it, was established, and the president of the association has been heard to declare that if the example is only followed domestic service will be revolutionised. Who shall doubt it?

### CINDERELLA—(*continued*).

AN EXAMINING AND CERTIFYING CENTRE.

London, in fact, is following this excellent lead. A National Council for Domestic Studies has established itself, with headquarters at Hastings House, Norfolk Street, Strand, and proposes to examine and certify students who have attended at least three-quarters of an approved course, a minimum of 620 hours. Studies are to comprise science applied to cookery, and catering and the treatment and care of all kinds of domestic material, some knowledge of gas and electric lighting, and heating and repairs to mechanism, elementary plumbing, home nursing and domestic hygiene, thrift, and account-keeping. That is to say, the commonest duties of home service are to be referred to their source in the conditions of civilised human life, and both seen and performed as part of a great service to the community. Truly George Herbert might see the making divine of drudgery in a broader if not a brighter light if he were living now! How far this immediate effort will prosper must depend on the spirit in which it is taken up by the schools to which it is proposed. It is certainly on right lines, and those of us who have seen what "domestic economy lessons" [illegible] well-managed centre have already done will not be [illegible]osed to call it Utopian.

